# A new enigmatic Late Miocene mylodontoid sloth from northern South America

**DOI:** 10.1098/rsos.140256

**Published:** 2015-02-25

**Authors:** Ascanio D. Rincón, H. Gregory McDonald, Andrés Solórzano, Mónica Núñez Flores, Damián Ruiz-Ramoni

**Affiliations:** 1 Instituto Venezolano de Investigaciones Científicas (IVIC), Laboratorio de Paleontología–Centro de Ecología, Km 11 de la Carretera Panamericana, Edo. Miranda. Aptdo. 21.827, Caracas Cod. Postal 1020-A, Venezuela; 2 Museum Management Program, National Park Service, 1201 Oakridge Drive, Fort Collins, CO 80525, USA

**Keywords:** Venezuela, *Eionaletherium tanycnemius*, Urumaco Formation, Mylodontoidea

## Abstract

A new genus and species of sloth (*Eionaletherium tanycnemius* gen. et sp. nov.) recently collected from the Late Miocene Urumaco Formation, Venezuela (northern South America) is herein described based on a partial skeleton including associated femora and tibiae. In order to make a preliminary analysis of the phylogenetic affinities of this new sloth we performed a discriminate analysis based on several characters of the femur and tibia of selected Mylodontoidea and Megatherioidea sloths. The consensus tree produced indicates that the new sloth, *E. tanycnemius*, is a member of the Mylodontoidea. Surprisingly, the new taxon shows some enigmatic features among Neogene mylodontoid sloths, e.g. femur with a robust lesser trochanter that projects medially and the straight distinctly elongated tibia. The discovery of *E. tanycnemius* increases the diversity of sloths present in the Urumaco sequence to ten taxa. This taxon supports previous studies of the sloth assemblage from the Urumaco sequence as it further indicates that there are several sloth lineages present that are unknown from the better sampled areas of southern South America.

## 2. Introduction

South America was an island continent through most of the Cenozoic until the establishment of the Panamanian land bridge connecting Central and South America approximately 2.8 Ma [1–3]. The isolation of South America as an island continent resulted in the evolution of a number of distinctive endemic linages of mammals. One of these is the Xenarthra, which today includes anteaters (Vermilingua), armadillos (Cingulata) and sloths (Phyllophaga) [4].

Within the Xenarthra, sloths are an extremely diverse lineage in terms of the number of species (more than 90 named genera) [5,6], a wide range of body sizes and a diversity of locomotor and feeding adaptations, which is reflected in the variety of habitats in which they lived [4,7–9].

The oldest remains of sloths come from the Early Oligocene (Tinguirirican SALMA) of Chile [10]. Sloths became abundant during the Late Oligocene (Deseadan SALMA), based mainly on records from Argentina and Bolivia, and by that time are represented by several distinct lineages [9,11,12]. Currently, the greatest diversity of sloths is documented for the Late Miocene and the Pleistocene [5]. Whether these two intervals are merely artefacts of the number of known sites or actually reflect periods of evolutionary diversification is not known at this time.

Early Neogene vertebrate sites in northern South America are limited [13]. The earliest record of sloths in northern South America probably comes from the Early Miocene (Burdigalian age) Castillo Formation [14,15], and from the ‘Early’ Miocene of Rio Yuca (*Pseudoprepotherium venezuelanum* Collins 1934 [16]) of Venezuela. Unfortunately, the age of the latter taxon remains unclear [15].

Northern South American Neogene sloths are found in exposures along the Acre River and its tributaries in Brazil and Peru. With a Late Miocene–Pliocene age and nine known taxa, the Acre fauna shows affinities with both northern and southern sloth faunas [17]. Another diverse northern South American sloth assemblage comes from the Middle Miocene of La Venta, Colombia, and includes a diverse sloth fauna from which at least eight or nine species from the Megatheriidae, Nothrotheriidae, Megalonychidae and Mylodontidae have been recovered [18,19]. The other major locality in northern South America with a diverse sloth fauna is the Urumaco sequence, Venezuela [14,20–23]. The Urumaco sequence includes three formations (Socorro, Urumaco and Codore) that were deposited from the Middle Miocene to Early Pliocene [23–27]. They represent a complex of marginal and near shore coastal environments (including near shore marine, deltaic system and fluvial settings without marine influence) [27–29]. The recognized sloths from the Urumaco sequence include nine species [14,20–23], but so far only four sloths have been reported from the Late Miocene Urumaco Formation, *Urumaquia robusta* Carlini, Scillato-Yané & Sánchez 2006, *Urumacotherium garciai* Bocquentin-Villanueva 1984, *Mirandabradys urumaquensis* Carlini, Brandoni & Sánchez-Villagra 2006 and *Bolivartherium urumaquensis* (Linares 2004). The sloth assemblages of both La Venta and Urumaco include very basal sloths and the earliest representatives of new lineages, as well as clades unknown from southern South America [18,22,30].

Intensive palaeontological fieldwork recently carried out by the Laboratory of Paleontology of IVIC in previously unexplored areas of the Urumaco Formation have resulted in the discovery of a new mylodontid sloth. The purpose of this paper is to provide a detailed morphological description of this new taxon, document its unusual morphology and discuss some aspects of its palaeobiology. Owing to the limited part of the skeleton recovered of this new taxon, in order to provide a preliminary understanding of its broader relationships to other sloths we provide a phylogenetic hypothesis based on only postcranial features of some North and South American sloths.

## 3. Geological setting

**Figure 1.**
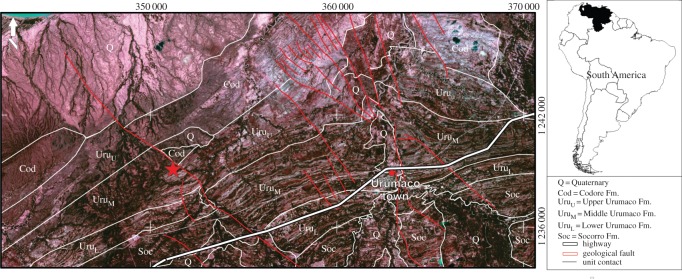
Geological map of the Urumaco sequence, northern South America. The white star shows the exact provenance of the new mylodontoid taxon: the middle member of the Urumaco Formation (Late Miocene), Buchivacoa Municipality, Falcón State, northwestern Venezuela.

The Urumaco sequence outcrops in the northwestern part of Falcón State, Venezuela and as defined here includes the Socorro, Urumaco and Codore Formations (figure 1). Since the initial fieldwork in this area by Bryan Patterson in 1972, this region has produced a diverse vertebrate fauna with over 88 described taxa [14]. The mammalian fauna of the Urumaco sequence includes a variety of sloths representing two families, Mylodontidae (*Mirandabradys* Carlini, Brandoni & Sánchez-Villagra 2006 represented by *Mirandabradys socorrensis* Carlini, Brandoni & Sánchez-Villagra 2006, *Mirandabradys zabasi* Carlini, Brandoni & Sánchez-Villagra 2006 and *M. urumaquensis*, *Bolivartherium* Carlini, Brandoni & Sánchez-Villagra 2006 represented by two species (*Bolivartherium codorensis* (Linares 2004) and *B. urumaquensis*) and *U. garciai*) and Megatheriidae (*U. robusta* and *Proeremotherium eljebe* Carlini, Brandoni & Sánchez-Villagra 2006).

The Urumaco Formation consists of a complex intercalation of medium- to fine-grained sandstone, organic-rich mudstone, coal, shale and thick-bedded coquinoidal limestone with abundant mollusc fragments [27]. The dominant palaeoenvironment during the deposition of the sediments of the Urumaco Formation is still unclear. According to Díaz de Gamero & Linares [28] and Hambalek *et al.* [29], the deposition of the Urumaco Formation occurred in a complex of marginal and near coastal environments. Quiroz & Jaramillo [27] suggests, based on the foraminifera, that the formation was probably deposited in a prograding strand plain–deltaic complex during the Late Miocene. The recovery of several terrestrial mammals in this Formation permitted its assignment to the Middle to Late Miocene [21]. The Urumaco Formation is informally divided into three members, lower, middle and upper. The fossils described here were collected from the middle member of the Urumaco Formation, at the ‘Charlie’ locality (11°11′45.8′′ N; 70°21′53.9′′ W). The shales found in this member represent deposition of low-energy suspension fallout on the shelf and prodelta [27].

## 4. Material and methods

The holotype specimen described here is housed at the Instituto Venezolano de Investigaciones Científicas (IVIC) in Caracas, Venezuela. All measurements are in millimetres and were taken with a digital calliper. The comparison of the newly described taxon is based principally on the other sloths from the Urumaco sequence.

### 4.1 Dataset

In order to estimate the broader phylogenetic context of the new sloth described herein, we developed a dataset that includes 24 characters based on the femur and tibia (see electronic supplementary material). We included 21 members of several lineages of South and North American sloths within the Mylodontidae, Megalonychidae, Megatheridae and Nothrotheridae that range in age from the Oligocene to Pleistocene. The character state assignments for the postcranial skeleton of the 21 taxa used in this study were based upon direct observation of specimens and information obtained from the primary literature.

### 4.2 Search methods

The dataset was analysed using the TNT 1.1 software [31]. All characters were treated as non-additive (unordered); gaps were treated as missing. The characters were analysed using ‘implied weights’ methodology with *k*=3. The heuristic parsimony analysis of 1000 replicates was performed using the ‘traditional search option’ [31]. The swapping algorithm used was tree bisection reconnection (TBR), with 10 trees saved per replication, collapsing the trees after each search. To measure node stability, we used the frequency differences (GC) arising from symmetric resampling (*p*=33) based on 1000 replicates. The outgroup taxon is the North American megalonychid *Megalonyx jeffersoni* Desmarest 1822, as the postcranial skeleton of this taxon is well known and possesses a distinct morphology of the femur and tibia compared with mylodonts. In order to elaborate an illustrative final tree, the obtained consensus tree was optimized with the results of the common synapomorphies, the common character modules and the support values. The present analysis is not meant as a comprehensive phylogenetic study (as e.g. [11]), it is merely to illustrate the broader relationships of the new taxon described to other known taxa using the parts of the skeleton available.

### 4.3 Body mass

To calculate the body mass of the specimens, we used the predictive regression equation derived from measurements of the femur derived from extant mammals developed by Scott [32]:

log mass=3.4855×log⁡fl−2.9112,

where fl is the femur length [32].

### 4.4 Institutional abbreviations

IVIC-P: Colección de Paleontología, Instituto Venezolano de Investigaciones Científicas, Caracas, Venezuela; MCN: Museo de Ciencias, Caracas, Venezuela.

## 5. Systematic palaeontology

Xenarthra Cope, 1889

Phyllophaga Owen, 1842

Mylodontoidea Gill, 1872

 *Eionaletherium tanycnemius* new genus and species

### 5.1 Etymology

 *Eion* (Greek, feminine)—shore; *ale* (Greek, feminine)—wanderer; *therium* (Greek)—beast. The shore wandering beast is in reference to the palaeoenvironment inferred for the Urumaco Formation. *Tanycnemius*—Greek for long leg in reference to the unusually long tibia compared with other sloths. *Tany* (Greek) is long or stretched out and *cnemius* (Greek, feminine)—the part of the leg between the knee and ankle.

### 5.2 Holotype

IVIC-P-2870: both femora, a complete right tibia and fibula, proximal and distal left tibia, some vertebrae, fragments of both scapulae, a very fragmented astragalus and many rib fragments were all found associated within an area of 2 m^2^, associated only with crocodiles and turtles and with remains of other mammals present, so they are considered to represent a single individual.

### 5.3 Type locality and horizon

Northwestern of Falcón state, Urumaco desert, Buchivacoa municipality (11°11′45.8′′ N; 70°21′53.9′′ W). Urumaco sequence, middle member of Urumaco Formation, Late Miocene (figure 1), probably equivalent to the Chasicoan-Huayquerian SALMAs [26].

### 5.4 Diagnosis

A medium to large mylodontoid, *E. tanycnemius* presents the following unique character combination that distinguishes it from other members of the Mylodontoidea or Megatherioidea: diaphysis of the femur slightly curved; shallow valley between the femur head and the greater trochanter; lesser trochanter robust, caudally and medially projected; third trochanter not projecting from the diaphysis of the femur relative to the lateral margin of the greater trochanter; proximal end of the femur broader than the distal end; ectepicondyle and entepicondyle robust and projecting laterally and medially, respectively; tibia straight, with massive diaphysis, and distinctively more elongated than any other Neogene mylodontoid; length ratio of tibia/femur around 0.87; fibula and tibia proximally and distally unfused.

### 5.5 Description and comparison

 *Eionaletherium tanycnemius* is larger than *Pseudoprepotherium confusum* Hirschfeld 1985 or *P. venezuelanum*, but is smaller than the other Urumaco mylodonts, *Mirandabradys* spp., *U. garciai* and *B. urumaquensis* (see the following discussion).

**Figure 2.**
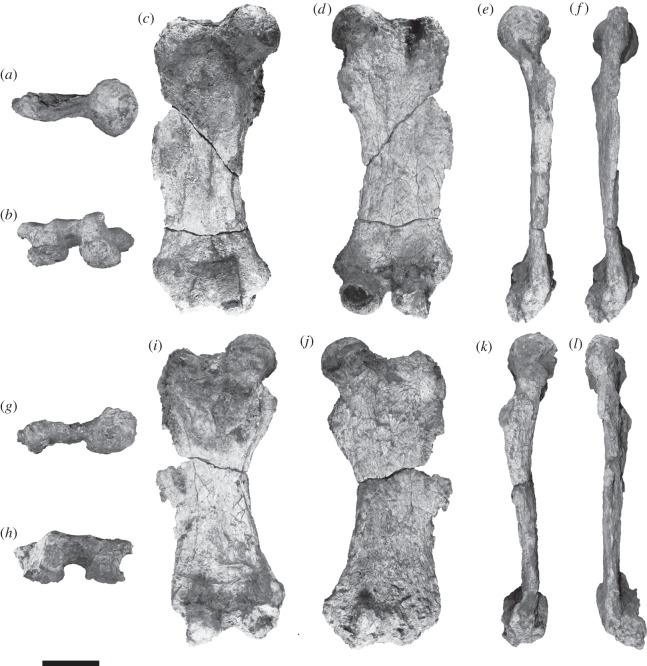
*Eionaletherium tanycnemius* gen. et sp. nov., IVIC-P-2870 (holotype). Right and left associated femora. Right femur in cranial (*a*), distal (*b*), posterior (*c*), anterior (*d*), medial (*e*), and lateral (*f*) views. Left femur in cranial (*g*), distal (*h*), posterior (*i*), anterior (*j*), medial (*k*) and lateral (*l*) views. Scale bar, 10 cm.

The femur (figure 2) exhibits the strong antero-posterior flattening seen in many of the Late Neogene mylodontoids sloths, unlike megatherioids in which the shape of the transverse diaphysis is cylindrical to oval. Both sides of the femur of *E. tanycnemius* are curved, whereas in *Mirandabradys* spp., and to a lesser degree in *P. venezuelanum* and *P. confusum*, the lateral side of the diaphysis of the femur is curved while the medial side is straight. As in most mylodonts the long axis of the diaphysis of the femur of *E. tanycnemius* slopes medially relative to the distal part of the femur as described by McDonald *et al*. [33]. The diaphysis of the femur of *E. tanycnemius* has less torsion than *Mirandabradys* spp., so the shaft appears columnar or straight. The diaphysis of the femur of *Mirandabradys* spp. is straight from the distal end to the third trochanter, but then becomes rotated medially at an angle relative to the shaft of 25° as in *P. venezuelanum* and *P. confusum*. In *E. tanycnemius*, the curvature of the femur is homogeneous and lacks any abrupt change in the axis of the diaphysis. The femur of *E. tanycnemius* has a clearly demarcated neck and in this feature resembles *M. socorrensis*. The valley between the femur head and the greater trochanter is very shallow in *E. tanycnemius*. The femur head is directed nearly to the medial side. The femur head of *E. tanycnemius* is larger than that of *Mirandabradys* spp. but is similar in size to that of *Bolivartherium* (*sensu lato*).

In *E. tanycnemius* the greater trochanter is below the plane of the femur head as in *M. socorrensis*, *M. urumaquensis* and *U. garciai* but differs from *M. zabasi*, *P. venezuelanum* and *B. urumaquensis*, in which the greater trochanter extends proximally to the level of the head of the femur, and from *P. confusum*, in which the greater trochanter is above the head of the femur. The greater trochanter is much more massive in *B. urumaquensis* than in *E. tanycnemius*. In *E. tanycnemius*, the lesser trochanter is very robust, projects medially and caudally, and is positioned directly below the femur head. In *B. urumaquensis*, the lesser trochanter is robust and also medially placed, but it is located along the medial border of the diaphysis, whereas in *Mirandabradys* spp., *P. venezuelanum*, *P. confusum* and *Urumacotherium* the lesser trochanter is smaller.

In *E. tanycnemius* the third trochanter is relatively small and located in the same plane as the greater trochanter and the lateral ectepicondyle along the lateral border of the diaphysis, as apparently occurs in *Mirandabradys* spp., and differs from *B. urumaquensis* and *P. confusum*, in which the third trochanter is larger, more developed and positioned more laterally and projects laterocaudally. The third trochanter is located slightly above the midpoint of the femur diaphysis in *E. tanycnemius*, slightly below the midpoint of the diaphysis in *Mirandabradys* spp. and *P. confusum* [18,22], and in the midpoint of the diaphysis in *B. urumaquensis* and *P. venezuelanum*.

In *E. tanycnemius* as in *U. garciai* the proximal end of the femur is broader than the distal end and differs from *Mirandabradys* spp., *B. urumaquensis*, *P. venezuelanum* and *P. confusum* in which the middle of the proximal end of the diaphysis is wider than the distal end of the femur. The ectepicondyle and entepicondyle of the femur of *E. tanycnemius* are more robust and project more laterally and medially, respectively, than in other Neogene mylodonts, *Mirandabradys* spp., *P. venezuelanum*, *P. confusum*, *B. urumaquensis* and especially more than in *Urumacotherium*. The patellar facet of the femur in *E. tanycnemius* is wider than long, compared with *M. urumaquensis* in which it is longer than wide, and differs from *M. socorrensis* in which the patellar facet is more rectangular, and in *E. tanycnemius* it is larger than that of *M. zabasi*. The patellar and condylar surfaces are connected, similar to other mylodonts. The medial and lateral condyles are asymmetrical in *E. tanycnemius*, while the medial and lateral condyle lengths (*sensu* [34]) are similar to that of *M. zabasi*. By contrast, the length of the lateral condyle is only 65–75% of the size of the medial condyle in *M. socorrensis*, *M. urumaquensis*, *P. venezuelanum*, *P. confusum*, *B. urumaquensis* and *U. garciai*.

**Figure 3.**
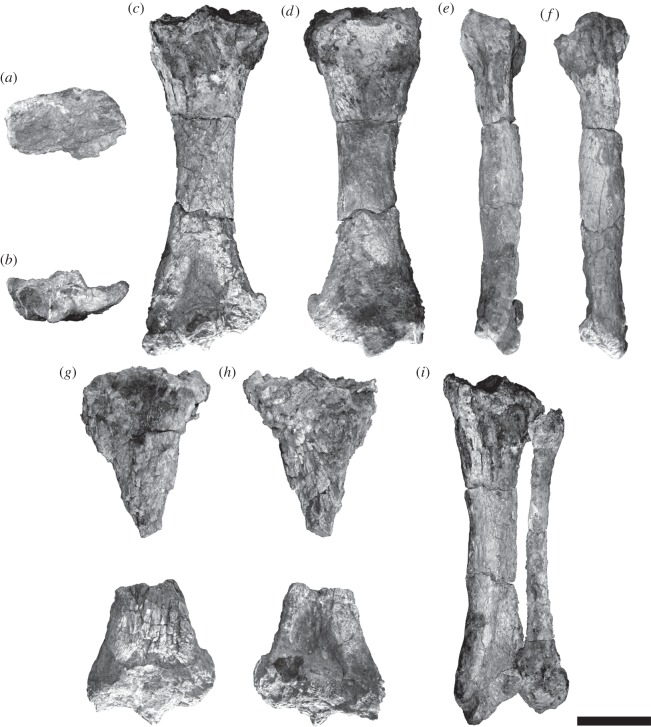
*Eionaletherium tanycnemius* gen. et sp. nov., IVIC-P-2870 (holotype). Right and left associated tibiae and fibulae. Right tibia in cranial (*a*), distal (*b*), posterior (*c*), anterior (*d*), lateral (*e*) and medial (*f*) views. Distal and proximal fragments of left tibia in anterior (*g*) and posterior (*h*) views. Right tibia and fibula disposition in postero-lateral view (*i*). Scale bar, 10 cm.

**Figure 4.**
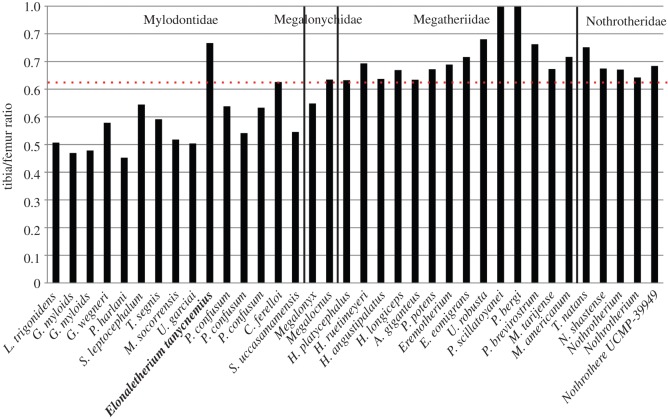
Graph of the tibia/femur ratios in some sloths, showing the remarkable difference between *Eionaletherium tanycnemius* gen. et sp. nov. and other sloths, and especially other mylodonts (see electronic supplementary material for details).

 *Eionaletherium tanycnemius* is clearly distinguished from other sloths, particularly other members of the Mylodontoidea, by its elongated tibia (figure 3). The ratio of the length of the tibia to the femur is greater than any other known mylodontoid sloth, with the tibia reaching 87% of the length of the femur, while in other mylodont sloths the length of the tibia ranges from 45% (*Paramylodon* Brown 1903) to 73% (*Chubutherium ferelloi* Cattoi 1962) of the length of the femur (figure 4; see electronic supplementary material for details). Generally, the ratio of the length of the tibia to the femur is less than 0.73 in mylodonts and megalonychids, while in nothrotheres and megatheres the ratio is above 0.73 (figure 4).

The diaphysis of the tibia in *E. tanycnemius* is straight (curved in *M. socorrensis*). The contour of the proximal epiphysis of the tibia is elliptic in cranial view (figure 3*a*), while the contour of the distal epiphysis is wider than longer (figure 3*b*). The proximal and distal epiphyses have approximately the same width in anterior view. The tibia is proximally and distally deformed, resulting in a distal epiphysis antero-posteriorly flattened and proximal epiphysis laterally flattened, while the diaphysis is massive. The proximal condylar facets are unfortunately poorly preserved in both tibiae. What is preserved of the medial condylar facet indicates it is more posteriorly placed compared with other sloths. The medial condylar facet is concave, whereas the lateral condylar facet is convex. The lateral condyle is sub-triangular in shape and smaller than the medial condyle. Both condylar facets are separated by the intercondylar eminence which is less pronounced than in *P. confusum*.

In the distal epiphysis, the medial malleolus and the inferior tibiofibula joint form an angle of 25–30° with the diaphysis of the tibia with the medial malleolus more distally placed than the inferior tibiofibula joint, unlike *P. confusum* in which that angle is smaller than 10°. On the lateral side of the distal epiphysis there is a well-developed tendonal groove which apparently does not exist in *P. confusum*.

The tibia and fibula are not fused distally or proximally in *E. tanycnemius* similar to *P. confusum*, but unlike *M. socorrensis* in which the tibia and fibula fuse, however whether this also occurs in other species of *Mirandabradys*, *P. venezuelanum* and *Bolivartherium* is currently unknown. The head of the fibula is sub-triangular in shape. The distal end of the fibula is more massive than the proximal end.

Although they are extremely poorly preserved, it is possible to see that the astragalus is quadrangular in cranial view. Although the astragalar facet of the tibia is deformed, the preserved portion suggests that the odontoid processes of the astragalus project more cranially than in *P. confusum*.

## 6. Phylogenetic affinities

Previous phylogenetic hypotheses about the relationships among extinct sloths using cladistic methods are limited and have been mostly based on craniodental features (e.g. [11,33,35,36]), and to a lesser extent have included both cranial and postcranial features [37]. The hypothesis presented here on the phylogenetic position of *E. tanycnemius* is only based on characters of the femur and tibia, so cannot be considered to represent a comprehensive phylogenetic study that refines our understanding of the phylogeny of these sloths. It does, however, serve as a useful tool to illustrate the broader relationships of *E. tanycnemius* to other known taxa until additional material such as the skull and dentition is recovered which will permit a refinement of this first approximation of its relationships. This also constitutes the first attempt to elucidate the affinities of several taxa based on parts of the postcranial skeleton, and for the first time *P. venezuelanum*, *U. garciai*, *C. ferelloi*, *Mirandabradys* spp. and *B. urumaquensis* are included in a phylogenetic analysis.

**Figure 5.**
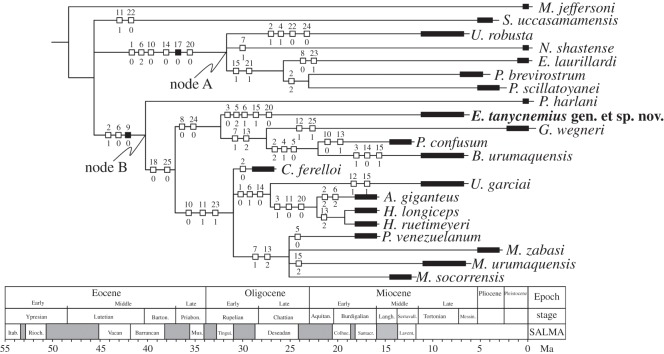
Strict consensus of 13 MPTs, found by tree bisection reconnection (CI=0.378; RI=0.630). The node at A represents the Megatheroidea and at B the Mylodontoidea.

We recovered six most parsimonious trees (MPTs) with a TBR score of 9.167 for the TNT analysis. These trees have a consistency index (CI) of 0.373 and a retention index (RI) of 0.620. The consensus tree is shown in figure 5. All of the most parsimonious hypotheses that emerged from this analysis place the new taxon, *E. tanycnemius*, at the base of a clade that includes *Glossotherium wegneri* Spillmann 1931, *P. confusum* and *B. urumaquensis.* *Glossotherium* is a member of the Mylodontidae while the other two taxa are usually placed in the Mylodontoidea [5].

At higher levels of resolution the consensus tree shows two apparently well-defined clades (figure 5). The first node (node A) is supported by two equivocal synapomorphies: valley between the femur head and the greater trochanter shallow (2^1^); proximal end of the femur broader than the distal end (6^0^); and one unequivocal synapomorphy: connection between patellar and condylar surfaces, connected (9^0^). The node A includes: *E. tanycnemius*, *G. wegneri*, *Mirandabradys* spp., *B. urumaquensis*, *P. venezuelanum*, *P. confusum*, *C. ferelloi*, *U. garciai*, *Paramylodon harlani* Owen 1840, *Hapalops ruetimeyeri* Ameghino 1891, *Hapalops longiceps* Scott 1901 and *Analcimorphus giganteus* Ameghino 1894.

The node B is supported by the following five equivocal synapomorphies: femur diaphysis shape straight (1^0^); proximal end of the femur narrower than the distal end (6^2^); fovea capitis present (10^0^); femur diaphysis cylindrical to oval in transverse shape (14^0^); tibia and fibula proximally and distally unfused (20^0^); and one unequivocal synapomorphy: distal condyles of the femur equal in size (17^0^). The node B suggests a close relationship among *Eremotherium laurillardi* Lund 1842, *Nothrotheriops shastense* Sinclair 1905, *U. robusta*, *Pyramiodontherium scillatoyanei* De Iuliis, Ré & Vizcaíno 2004 and *Pyramiodontherium brevirostrum* Carlini, Brandoni, Scillato-Yané & Pujos 2002.

## 7. Discussion

The most parsimonious hypotheses (figure 5), plus several features observed in the postcranial elements of *E. tanycnemius* (e.g. femur diaphysis curved and strong antero-posterior flattening; patellar and condylar surfaces connected), strongly suggest that it be considered an unequivocal member of the Mylodontoidea, and probably a Mylodontidae (see the following discussion). Although *E. tanycnemius* show some enigmatic features similar to the Megatheriidae and Nothrotheriidae (e.g. ratio of the length of the tibia to the femur is greater than 0.73), we considered this last feature to be convergent with these other sloths and reflects an adaptation to a particular palaeobiological setting (see the following discussion).

Taxa grouped into the node A (figure 5) include (besides *E. tanycnemius*) *Glossotherium*, *Paramylodon* and *P. confusum*, considered based on craniodental evidence to be members of the Mylodontoidea [11]. However, the phylogenetic position of the Santacrucian sloths *H. ruetimeyeri*, *H. longiceps* and *A. giganteus* within our tree is contradictory to their phylogenetic relationships based on cranial and dental characters. In this analysis, they are nested within the Mylodontoidea contrary to their inclusion within the Megatherioidea [5]. Although Gaudin [11] placed *Hapalops* and *Analcimorphus* at the base of the Megatherioidea, he did note that these sloths are quite distinct from the post-Santacrucian lineages [37]. *Hapalops* is often considered as a ‘typical primitive sloth’ [37,38]. The cladistic analysis of De Iuliis [38] which included cranial and postcranial elements placed *H. longiceps* in an unresolved position outside the Megatherioidea. Based on cranial features, we agree that *H. ruetimeyeri*, *H. longiceps* and *A. giganteus* are more closely related to primitive Megatherioidea than to members of the Mylodontoidea, but it is remarkable that the general morphology of their femur and tibia resembles those of later Mylodontoidea. In the node B (figure 5), except for *Nothrotheriops* Hoffstetter 1954 (Nothrotheriidae), all of these other genera are formally considered members of the Megatheriidae [5,11]. The two families Megatheriidae and Nothrotheriidae are included in the Megatherioidea.

The position of *Simomylodon* Saint-André, Pujos, Cartelle, De Iuliis, Gaudin, McDonald and Mamani-Quispe 2010 in the consensus tree (figure 5) remains unresolved owing to its position as the sister group of both the Mylodontoidea (node A) and Megatherioidea (node B) despite *Simomylodon* being described as a Mylodontinae [39].

So, although we include taxa not used by Gaudin [11] in his study, our consensus tree generally agrees with the general relationships of genera that the two studies do have in common. This suggests that it is possible to achieve a relatively clear phylogenetic signal based on the postcranial skeleton similar to that obtained from an analysis of the cranial elements [11].

The elongation of the tibia and fibula of *E. tanycnemius* relative to the length of the femur is greater than in any other mylodontoid (figure 4). In this regard, *E. tanycnemius* is convergent with the nothrothere sloth, *Thalassocnus* de Muizon and McDonald 1995 from Peru and Chile. *Thalassocnus* is one of the more aberrant known sloths and is the only xenarthran proposed to be aquatic or semiaquatic [40–44]. With five recognized species, and a biochron restricted to the Late Miocene–Early Pliocene of southern Peru and northern Chile, *Thalassocnus* lived at the same time as *E. tanycnemius*. Among its many unique features of the skeleton *Thalassocnus* is distinguished by having a tibia which is proportionally longer, about 85% of femoral length [40]. This elongation of the tibia is interpreted as an adaptation for locomotion in an aquatic environment [40].

While speculative at this time based on the small percentage of the skeleton recovered, those parts that are preserved raise the interesting possibility that this new mylodont, *E. tanycnemius*, may have independently evolved the ability to live in a near shore aquatic environment based on the types of sediments consisting of alternating near shore marine and terrestrial environments in the Urumaco sequence. However, other features of the skeleton of *E. tanycnemius* do not support this conclusion. For example, the bone compactness (BC) in *E. tanycnemius* for the first rib is BC=0.66 (23.5 mm/15.4 mm) and in the second rib it is BC=0.77 (18.1 mm/13.9 mm). These values are lower in comparison with *Thalassocnus*, *Thalassocnus antiquus* de Muizon, McDonald, Salas & Urbina 2003 (the earliest species) having a value of 0.785 and *Thalassocnus carolomartini* McDonald & Muizon 2002 (the youngest species) a value of 0.955 [45]. It should be noted that the BC in *Thalassocnus* increased gradually over a short geological time span of about 5 Myr, which is considerably less than that recorded at Urumaco but does reflect a shift in the animal's increasing utilization of an aquatic habitat [45].

The fovea capitis femoris is absent in *E. tanycnemius*, indicating a weak abduction at the femoroacetabular joint necessary for a powerful stroke of the hind limb that would aid in swimming. While the relatively longer tibia with respect to the femur in *E. tanycnemius* is common in aquatic mammals, the low value of BC and the absence of fovea capitis femoris on the femur are inconsistent with an interpretation of an aquatic lifestyle as seen in other mammals. The feature of the skeleton more indicative of a terrestrial lifestyle for *E. tanycnemius* is the lack of the fovea on the head of the femur.

One of the greatest skeletal differences between Miocene and Pleistocene mylodonts is in the tremendous shortening and torsion of the tibia in the latter. The result is that the lateral femoral facet projects beyond the margin of the shaft and this shifts the attachment of the patellar ligament laterally so the anterior face of the tibia is directed more to the inner side [18]. *E. tanycnemius* is similar to other Miocene mylodonts and differs from the Pleistocene taxa in that the tibia lacks this torsion and the lateral femoral articular surface is positioned directly over the shaft of the tibia.

Along with *E. tanycnemius*, other mylodontoids from the Urumaco Formation include *M. urumaquensis*, *B. urumaquensis* and *U. garcai*. It should be noted that Bocquentin-Villanueva and Sánchez-Villagra & Aguilera [20,46] list *U. garcai* as a megathere, and Bocquentin-Villanueva [20] even considered *Urumacotherium* to belong to the subfamily Prepotheriinae. We disagree with this assignment and tentatively concur with Negri & Ferigolo [47] who placed it in the Mylodontidae, although in a new subfamily, Urumacotheriinae. Their interpretation of the morphology of the lower fourth molariform with the reduction of the posterior lobe so that the bilobation of this tooth seen in other mylodonts is not as pronounced is intriguing and requires further investigation. *Bolivartherium* is placed in the subfamily Lestodontinae, reflecting features of its skeleton that led to the original assignment of the taxon to the genus *Lestodon* Gervais 1855 by Linares [21]. *Mirandabradys* was placed in the Mylodontoidea but was not formally assigned to a family. Based on the result of our phylogenetic analysis (figure 5), we consider it and also *E. tanycnemius* tentatively as members of the Mylodontidae. Given the limited amount of material of *E. tanycnemius* and its unusual morphology compared with other mylodonts, we follow the example of Hoffstetter [48] with *Pseudoprepotherium* of waiting until cranial material is found that might permit a better resolution of its relationship within the Mylodontidae and assigning it to a subfamily.

It should also be noted that the family Mylodontidae is in serious need of a full taxonomic review and a comprehensive phylogenetic study. McKenna & Bell [5] recognized only two subfamilies within the family Mylodontidae, Mylodontinae and Lestodontinae, excluding the Scelidotheriinae, as they considered the scelidotheres a separate family. As none of the taxonomic groups within their work has an associated diagnosis with regard to the characters used to define them, any attempt to refine the systematics of the mylodont sloths will require a close examination and reassessment of all the currently known taxa. The subfamily Urumacotheriinae proposed by Negri & Ferigolo [47] indicates the initial recognition of multiple different lineages within the Mylodontidae besides the traditional subfamilies. The morphological diversity within the Mylodontidae seen in the mandibles and lower dentition alone is clearly indicated by Rinderknecht *et al*. [49], although no formal taxonomic groups were proposed.

So far the earliest species of sloth in northern South America is *P. venezuelanum*, recovered from the Río Tucupido in Portuguesa State, Venezuela [16]. The holotype of *P. venezuelanum* is an isolated femur, which is now unfortunately missing. This taxon was originally placed in *Prepotherium* Ameghino 1981 by Collins [16], but later Hoffstetter [48] recognized it was not that genus and proposed the name *Pseudoprepotherium* Hoffstetter 1961, making *P. venezuelanum* the genotypic species. Hoffstetter [48] indicated that the affinities of *P. venezuelanum* were unknown, and the discovery of skull material would be needed to resolve this issue. Hirschfeld [18] subsequently recognized a new sloth species from La Venta, Colombia, and assigned it to *Pseudoprepotherium* as *P. confusum*, based on cranial and postcranial elements. The cranial material available allowed her to determine it was a mylodont and she assigned it to the subfamily Mylodontinae [18]. Gaudin [11] considered *P. confusum* as a member of the Mylodontidae.

Among the lower-level relationships obtained by our phylogenetic analysis, it is interesting to note the close relationship between *P. venezuelanum* and *Mirandabradys* spp. based on the following equivocal synapomorphies: greater trochanter larger or closely equal in size to the head (7^1^); and greater trochanter position almost at same level as the femur head (13^2^). Although the original specimen of *P. venezuelanum* is currently missing, the illustration provided by Collins [16] is sufficient to determine the major features of its morphology, which include the synapomorphies previously mentioned plus a small lesser trochanter which resembles that of *Mirandabradys* spp. (and especially *M. zabasi* from the Codore Formation). We consider it a strong possibility that they may be the same taxa, however a more comprehensive comparative analysis is needed in order to confirm this hypothesis.

Another result of our analysis is the position of the two species of *Pseudoprepotherium* in the consensus tree (figure 5), which suggests that this may not be a monophyletic taxon. As previously suggested the type species, *P. venezuelanum*, appears to be more similar to *Mirandabradys* spp. Additionally, the result of our phylogenetic analysis suggests a closer relationship between *P. confusum* and *B. urumaquensis* (figure 5) as *P. confusum* groups with *B. urumaquensis* based on three equivocal synapomorphies: deep valley between the femur head and the greater trochanter (2^2^); third trochanter projects posteriorly (4^1^); and third trochanter position at the middle of the diaphysis (5^0^). It is possible that *P. confusum* does not belong to the genus *Pseudoprepotherium*. While this possibility is intriguing based on the available material used in this study, we are unable to confirm this hypothesis. We would also note in passing similarities in the skull of the other species of *Bolivartherium*, *B. codorensis*, with that of *Lestobradys sprechmanni* Rinderknecht, Bostelmann, Perea & Lecuona 2010 from the Huayquerian of Uruguay [49], and if this is the case it indicates linkages between Urumaco and southern South America in the Late Miocene.

Independent of any formal taxonomic assignment to subfamily, there is clear diversity within the morphology of the mylodontoid sloths at Urumaco (at least based on the comparable elements preserved), which consequently suggests there must have been diversity also in the palaeoecology of the different taxa. Since the femur is preserved for *E. tanycnemius* and all of the other mylodontoids from Urumaco, this permits us to make estimates of the body mass of each taxon based on the same skeletal element. While at the larger end of the size range for sloths (see electronic supplementary material for details) with an estimated body mass of 1098 kg, *E. tanycnemius* is still smaller than the other mylodontoid sloths from Urumaco, *Mirandabradys* (with an estimated body mass ranging about 1297 to 1868 kg), *Urumacotherium* (2108 kg) and *Bolivatherium* (1719 kg), making it similar in size to *U. robusta* (1025 kg). This indicates that the new taxa *E. tanycnemius* is one of the smaller sized sloths in the Late Miocene of the Urumaco sequence.

## 8. Conclusion

The combination of unusual features of *E. tanycnemius* (e.g. straight tibia and femur, elongated tibia, unfused fibula and tibia) are surprising, taking into account the other known mylodontids from the Neogene of northern South America (*Mirandabradys* spp., *P. venezuelanum* *P. confusum* and *Urumacotherium*). These other sloths have derived features such as the fusion of the tibia and fibula (as in *Mirandabradys* spp.) and shortening of the tibia (as in *P. confusum* and *Mirandabradys* spp.). Thus, *E. tanycnemius* retains some basal mylodontid features, or it is possibly extremely derived given that all other sloths have a short tibia relative to the length of the femur.

The most parsimonious hypotheses suggests that *E. tanycnemius* be considered a Mylodontoidea (and even Mylodontidae), as well as *Mirandabradys* spp., *P. venezuelanum* and *Urumacotherium*, thus increasing the diversity of mylodontoids in the Urumaco sequence. This new taxon might also represent a new lineage of mylodontoid sloths in northern South America that is currently unknown from the extensively prospected sites of southern South America [30]. It is clear that at the moment the southern portion of South America has been better studied than the northern Neotropical portion of the continent so our understanding of the early origins of the mylodontoid is geographically limited. Future studies in the Urumaco sequence, as well as in earlier localities (e.g. the Early Miocene Castillo Formation), will improve our knowledge of the origins and diversification of this group of sloths.

## Supplementary Material

1. Characters developed for the cladistics analysis that includes 25 characters based on characters based on the femur and tibia. 2. Character matrix using for the phylogenetic analysis. 3. TNT output with the strict consensus obtain for the cladistics analysis. 4. Measurements (in mm) of the femur and tibia length, ratio F/T, and body mass (in kg) of some sloths, with their respective reference. 5. References

## Data accessibility

All data are attached as electronic supplementary material. The published work and the nomenclatural acts it contains (new genus and species names) have also been registered in Zoobank under the following LSIDs (Life Science Identifiers): Original publication: 75117137-179A-4EE4-86F0-063CEC82A102. *Eionaletherium* new genus: 94F20411-5929-43B3-A39A-8A35CA26FA8B. *Eionaletherium tanycnemius* new species: F536AFB9-47F5-43EC-894D-3AB228FE04DB.
