# A new enigmatic Late Miocene mylodontoid sloth from northern South America

**DOI:** 10.1098/rsos.150138

**Published:** 2015-04-29

**Authors:** Ascanio D. Rincón, H. Gregory McDonald, Andrés Solórzano, Mónica Núñez Flores, Damián Ruiz-Ramoni

*R. Soc. open sci.* **2**, 140256. (Published online 25 February 2015). (doi:10.1098/rsos.140256)

This correction seeks to amend some mistakes in the character list developed for the cladistics analysis based on the femur and tibia, which were provided in the associated electronic supplementary material. Inadvertently, one character from the final list was omitted and, as a consequence, some citations in the main text must be corrected. In the Material and methods section, the number of characters used in the phylogenetic analysis should be **25** instead 24. In the Phylogenetic affinities section (last paragraph), please note the following changes (updates marked in bold):

The node B is supported by the following five equivocal synapomorphies: femur diaphysis shape straight (1^0^); proximal end of the femur narrower than the distal end (6^2^); fovea capitis present (10^0^); femur diaphysis cylindrical to oval in transverse shape (14^0^); **ratio of length of tibia/femur greater than or equal to 73% (20^0^)** and one unequivocal synapomorphy: **distal condyles of the femur equal in size (17^0^).**

The correct character list development for the phylogenetic affinities of *Eionaletherium tanycnemius* is as follows (with the update marked in bold):

Character 1. Femur diaphysis shape: straight (0); curved (1).
Character 2. Valley between the femur head and the greater trochanter: absent (0); shallow (1); deep valley (2).
Character 3. Lesser trochanter: well-developed, and caudally and medially directed (0); well-developed, and slightly caudally but more medially directed (1); poorly developed, and aligned with the diaphysis (2).
Character 4. Third trochanter: does not project from the diaphysis of the femur relative to the lateral margin of the greater trochanter (0); projects posteriorly (1).
Character 5. Third trochanter position: at the middle of the diaphysis (0); distal to the middle of the diaphysis (1); proximal to the middle of the diaphysis (2).
Character 6. Proximal end of the femur: broader than the distal end (0); same width as the distal end (1); narrower than the distal end (2).
Character 7. Greater trochanter size: longer than wide and smaller than the head (0); larger or closely equal in size to the head (1).
Character 8. Ectepicondyle and entepicondyle: robust and prominently projected laterally and medially, respectively (0); small and only project slightly (1).
Character 9. Connection between patellar and condylar surfaces: connected (0); separate (1).
Character 10. Fovea capitis: present (0); absent (1).
Character 11. Femur neck: well demarcated (0); not well demarcated (1).
Character 12. Femur head angle with respect to the diaphysis: around 80–120° (0); more than 160° (1).
Character 13. Greater trochanter position: below to the femur head (0); above the femur head (1); almost at same level as the femur head (2).
Character 14. Femur diaphysis transverse shape: cylindrical to oval (0); anteroposteriorly flattened (1).
Character 15. Patellar surface shape: shorter than wide (0); length and width similar (1); longer than wide (2).
Character 16. Fossa trochanteric: deep and short (0); shallower and longer (1).
Character 17. Size of the femur condyles: equal in size (0): medial larger than lateral (1).
**Character 18. Femur borders: both sides curved (0); medial side curved and lateral side convex (1).**
Character 19. Tibia diaphysis shape: straight (0); curved (1).
Character 20. Tibia/femur ratio elongation: greater than or equal to 73% of the femur length (0); less than 73% of femur length (1).
Character 21. Fibula and tibia connection: proximally and distally unfused (0); fused (1).
Character 22. Articular proximal surfaces of the tibia: positioned above the diaphysis (0); lateral articular surface displaced laterally (1).
Character 23. Distal fibular articulation for tibia: posterolateral (0); lateral (1).
Character 24. Proximal articular facets of the tibia: both concave (0); medial facet convex and lateral concave (1).
Character 25. Process odontoides angle of the astragalus: more than 90° (0); equal to 90° (1).

