# Supplementary material for: A new enigmatic Late Miocene mylodontoid sloth from northern South America
Source: R Soc Open Sci. 2015 Feb 25;2(2):140256. doi: 10.1098/rsos.140256 (PMC4448802; doi:10.1098/rsos.140256)
Supplement: 1. Characters developed for the cladistics analysis that includes 25 characters based on characters based on the femur and tibia. 2. Character matrix using for the phylogenetic analysis. 3. TNT output with the strict consensus obtain for the cladistics analysis. 4. Measurements (in mm) of the femur  [file rsos140256supp1.docx]

ELECTRONIC SUPPLEMENTARY MATERIAL FOR:

A new enigmatic late Miocene mylodontoid sloth from northern South America.

**Ascanio D. Rincón^1,^*, H. Gregory McDonald^2^, Andrés Solórzano^1^, Mónica Núñez Flores^1^ & Damián Ruiz-Ramoni^1^.**

^1^*Instituto Venezolano de Investigaciones Científicas (IVIC), Laboratorio de Paleontología–Centro de Ecología, Km 11 de la Carretera Panamericana, Edo. Miranda. Aptdo. 21.827, Cod. Postal 1020-A, Caracas, Venezuela.*

^2^*Museum Management Program, National Park Service, 1201 Oakridge Drive, Fort Collins, Colorado 80525, U.S.A.*

**The Electronic Supplementary Material includes:**

1. Characters developed for the cladistics analysis that includes 25 characters based on characters based on the femur and tibia.

2. Character matrix using for the phylogenetic analysis.

3. TNT output with the strict consensus obtain for the cladistics analysis.

4. Measurements (in mm) of the femur and tibia length, ratio F/T, and body mass (in kg) of some sloths, with their respective reference.

5. References

**1. Characters developed for the cladistics analysis that includes 24 characters based on characters based on the femur and tibia**.

Character 1.- Femur diaphysis shape: straight (0); curved (1).

Character 2.- Valley between the femur head and the greater trochanter: absent (0); shallow (1); deep valley (2).

Character 3.- Lesser trochanter: well-developed, and caudally and medially directed (0); well-developed, and slightly caudally but more medially directed (1); poorly-developed, and aligned with the diaphysis (2).

Character 4.- Third trochanter: does not project from the diaphysis of the femur relative to the lateral margin of the greater trochanter (0); projects posteriorly (1).

Character 5.- Third trochanter position: at the middle of the diaphysis (0); distal to the middle of the diaphysis (1); proximal to the middle of the diaphysis (2).

Character 6.- Proximal end of the femur: broader than the distal end (0); same width as the distal end (1); narrower than the distal end (2).

Character 7.- Greater trochanter size: longer than wide and smaller than the head (0); larger or closely equal in size to the head (1).

Character 8.- Ectepicondyle and entepicondyle: robust and prominently projected laterally and medially respectively (0); small and only project slightly (1).

Character 9.- Connection between patellar and condylar surfaces: connected (0); separate (1).

Character 10.- Fovea capitis: present (0); absent (1).

Character 11.- Femur neck: well demarcated (0); not well demarcated (1).

Character 12.- Femur head angle with respect to the diaphysis: around 80-120 degrees (0); >160 degrees (1).

Character 13.- Greater trochanter position: below to the femur head (0); above the femur head (1); almost at same level as the femur head (2).

Character 14.- Femur diaphysis transverse shape: cylindrical to oval (0); anteroposteriorly flattened (1).

Character 15.- Patellar surface shape: shorter than wide (0); length and width similar (1); longer than wide (2).

Character 16.- Fossa trochanteric: deep and short (0); shallower and longer (1).

Character 17.- Size of the femur condyles: equal in size (0): medial larger than lateral (1).

Character 18.- Tibia diaphysis shape: straight (0); curved (1).

Character 19.- Tibia/femur ratio elongation: ≥73% of the femur length (0); <73% of femur length (1).

Character 20.- Fibula and tibia connection: proximally and distally unfused (0); fused (1).

Character 21.- Articular proximal surfaces of the tibia: positioned above the diaphysis (0); lateral articular surface displaced laterally (1).

Character 22.- Distal fibular articulation for tibia: posterolateral (0); lateral (1).

Character 23.- Proximal articular facets of the tibia: both concave (0); medial facet convex and lateral concave (1).

Character 24.- Process odontoides angle of the astragalus: >90° (0); =90° (1).

**2. Character matrix using for the phylogenetic analysis. The data set was analyzed using the TNT 1.1 software [31].**

*Megalonyx_jeffersoni* 101001011-010101111101010

*Glossotherium_wegneri* 11201010-101210--00100001

*Paramylodon_harlani* 1120100101010100111101011

*Mirandabradys_socorrensis* 112110110010210010111110-

*Mirandabradys_urumaquensis* 112110110010012010-------

*Mirandabradys_zabasi* 112010110010210010-------

*Bolivartherium_urumaquensis* 121100100100201110-------

*Urumacotherium_garciai* 012--1010-11001-10-1-----

*Eionaletherium_tanycnemius* 1100210001000110100000000

*Pseudoprepotherium_venezuelanum* 112000110010210010-------

*Pseudoprepotherium_confusum* 1221001000001101100101000

*Chubutherium_ferelloi* 10201001--10010010-----1-

*Simomylodon_uccasamamensis* 102011011110010-110100011

*Hapalops_ruetimeyeri*  01102101-000200--01001110

*Hapalops_longiceps* 01102101-000200--0-00-110

*Analcimorphus_giganteus* 02100201-000000--01001110

*Eremotherium_laurillardi* 002012001000001-010011111

*Nothrotheriops_shastense* 0020121110010000011001011

*Urumaquia_robusta* 012112011000000-010000-01

*Pyramiodontherium_scillatoyanei* 022012011-0100110100110-1

*Pyramiodontherium_brevirostrum* 022012011-01001-010011-11

**3. TNT output with the strict consensus of the 6 MPTs, using character matrix previously described and the ‘implied weight’** **methodology with k=3.**

**
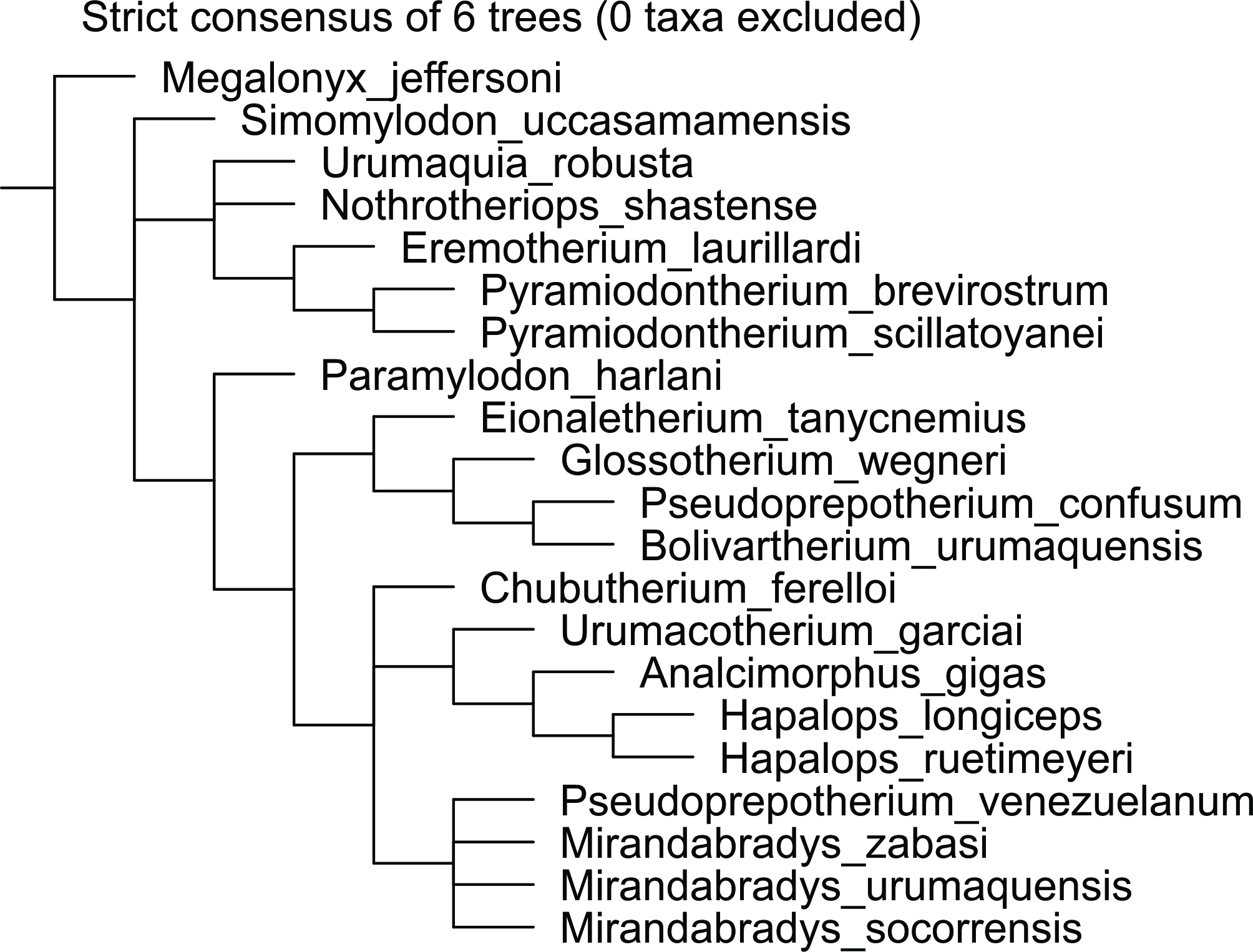
**

**4. Measurements (in mm) of the femur and tibia length, ratio F/T, and body mass (in kg) of some sloths, with their respective reference. The body mass (kg) was calculated using the equation development by Scott [32] as mentioned in the main text. The femur and tibia lengths were taken from distinct authors detailed in the reference.**

| Family | Taxa | Femur length | Tibia length | Ratio T/F | Body mass (kg) |
| --- | --- | --- | --- | --- | --- |
| Mylodontidae | *L. trigonidens*[23] | 730 | 370 | 0.51 | 3,832 |
|  | *G. myloides*[23] | 490 | 230 | 0.47 | 955 |
|  | *G. myloides*[23] | 460 | 220 | 0.48 | 766 |
|  | *G. wegneri*[50] | 472 | 273 | 0.58 | 838 |
|  | *P. harlani*[51] | 546 | 247 | 0.45 | 1,396 |
|  | *S. leptocephalum*[23] | 450 | 290 | 0.64 | 710 |
|  | *T. segnis*[23] | 443 | 262 | 0.59 | 672 |
|  | *M. socorrensis*[23] | 560 | 290 | 0.52 | 1,521 |
|  | *M. urumaquensis*[23] | 594 | - | - | 1,868 |
|  | *M. zebasi*[23] | 571 | - | - | 1,628 |
|  | *M. zebasi*[21] | 535 | - | - | 1,297 |
|  | *U. garciai*[23] | 615 | 310 | 0.50 | 2,108 |
|  | *E. tanycnemius* gen. et sp. nov. | 510 | 442 | 0.87 | 1,098 |
|  | *P. venezuelanun*[16] | 419 | - | - | 553 |
|  | *B. urumaquensis*[23] | 580 | - | - | 1,719 |
|  | *P. confusum*[23] | 470 | 300 | 0.64 | 826 |
|  | *P. confusum*[18] | 480 | 260 | 0.54 | 889 |
|  | *P. confusum*[18] | 484 | 307 | 0.63 | 915 |
|  | *C. ferelloi*[52] | 383 | 278 | 0.73 | 405 |
|  | *S. uccasamamensis*[39] | 325 | 177 | 0.55 | 227 |
| Megalonychidae | *Megalonyx*[42] | 617 | 400 | 0.65 | 2,132 |
|  | *Megalocnus*[42] | 286 | 210 | 0.73 | 146 |
| Megatheriidae | *Hapalops platycephalus*[53] | 168 | 123 | 0.73 | 23 |
|  | *H. ruetimeyeri*[53] | 150 | 119 | 0.79 | 15 |
|  | *H. angustipalatus*[53] | 190 | 140 | 0.74 | 35 |
|  | *H. longiceps*[53] | 212 | 163 | 0.77 | 51 |
|  | *A. giganteus*[53] | 218 | 160 | 0.73 | 57 |
|  | *P. potens*[53] | 298 | 230 | 0.77 | 169 |
|  | *Eremotherium*[42] | 711 | 561 | 0.79 | 3,495 |
|  | *E. eomigrans*[54] | 599 | 489 | 0.82 | 1,923 |
|  | *U. robusta*[22] | 500 | 440 | 0.88 | 1,025 |
|  | *P. scillatoyanei*[23] | 473 | 472 | 1.00 | 844 |
|  | *P. bergi*[23] | 515 | 514 | 1.00 | 1,136 |
|  | *P. brevirostrum*[23] | 580 | 500 | 0.86 | 1,719 |
|  | *M. tarijense*[23] | 528 | 408 | 0.77 | 1,239 |
|  | *M. americanum*[23] | 759 | 620 | 0.82 | 4,389 |
| Nothrotheriidae | *T. natans*[42] | 289 | 246 | 0.85 | 152 |
|  | *N shastense*[51] | 398 | 308 | 0.77 | 461 |
|  | *Nothrotherium*[42] | 231 | 178 | 0.77 | 69 |
|  | *Nothrotherium*[42] | 341 | 253 | 0.74 | 270 |

**4. References**

*16.* Collins RL. 1934. Venezuelan Tertiary mammals. Studies in Geology (The Johns Hopkins University) 11, 235–244.

*18.* Hirschfeld SE. 1985. Ground sloths from the Firasian La Venta fauna, with additions to the Pre-Friasian Coyaima fauna of Colombia, South America. Geological Sciences (University of California Publications) 128, 1–91.

*21.* Linares O. 2004a. Nuevos restos del genero *Lestodon* Gervais, 1855 (Xenarthra, Tardigrada, Mylodontidae), del Mioceno Tardío y Plioceno Temprano de Urumaco (Venezuela), con descripción de dos nuevas especies. Paleobiología Neotropical 2, 1–14.

*22.* Carlini AA, Brandoni D & Sánchez-Villagra MR. 2006a. First Megatherines (Xenarthra, Phyllophaga, Megatheriidae) from the Urumaco (late Miocene) and Codore (Pliocene) formations, Estado Falcón, Venezuela. J Syst Palaeontol 4, 269–278. http://dx.doi.org/10.1017/s1477201906001878.

*23.* Carlini AA, Scillato-Yané GJ & Sánchez R. 2006b. New Mylodontoidea (Xenarthra, Phyllophaga) from the middle Miocene–Pliocene of Venezuela. J Syst Palaeontol 4(3), 255–267. http://dx.doi.org/10.1017/s147720190600191x

*31.* Goloboff P, Farris J & Nixon K. 2008. TNT, a free program for phylogenetic analysis. Cladistics 24, 774–786. DOI: 10.1111/j.1096-0031.2008.00217.x

*32.* Scott KM. 1990. Postcranial dimensions of ungulates as predictors of body mass. In: Damuth J & MacFadden BJ (eds) Body Size in Mammalian Paleobiology: Estimation and Biological Implications. New York: Cambridge University Press, pp 331–335.

*39.* Saint-André PA, Pujos F, Cartelle C, De Iuliis G, Gaudin TJ, McDonald HG & Mamani-Quispe B. 2010. Nouveaux paresseux terrestres (Mammalia, Xenarthra, Mylodontidae) du Néogène de l’Altiplano bolivien. Geodiversitas 32, 255-306.

*42.* Muizon C de, McDonald HG, Salas R & Urbina M. 2004a. The youngest species of the sloth *Thalassocnus* and a reassessment of the relationships of the sloths (Mammalia: Xenarthra). J. Vert. Paleontol 24, 387–397. http://dx.doi.org/10.1671/2429a

*50.* Hoffstetter R. 1952. Les Mammifères Pléistocènes de la Républiqu del´Equateur: Mémoires de la Société Géologique de France, NouvelleSerie 31, 391 pp.

*51.* Stock CC. 1925. Cenozoic gravigrade edentates of western North America, with special reference to the Pleistocene Megalonychinae and Mylodontidae of Rancho la Brea. Carn. Inst Wash. Publ. 331: 1–206.

*52.* Cattoi N. 1962. Un nuevo Xenarthra del Terciario de Patagonia, *Chubutherium* *ferelloi*, gen. et sp. nov. (Megalonychoidea, Mylodontidae). Revista del Museo Argentino de Ciencias Naturales" Bernardino Rivadavia" Ciencias zoológicas 8(11): 123–134.

*53.* Scott WB. 1903. Mammalia of the Santa Cruz Beds, I. Edentata, II. Insectivora., III. Glires. Reports of the Princeton University Expeditions to Patagonia 1896-1899, V, 808 pp.

*54.* De Iuliis G & Cartelle C. 1999. A new giant megatheriine ground sloth (Mammalia: Xenarthra: Megatheriidae) from the late Blancan to early Irvingtonian of Florida. Zool J Linnean Soc 127: 495–515. DOI: 10.1111/j.1096-3642.1999.tb01383.x
